# Moxifloxacin releasing intraocular implant based on a cross-linked hyaluronic acid membrane

**DOI:** 10.1038/s41598-021-03605-0

**Published:** 2021-12-16

**Authors:** Dong Ju Kim, Mi-Young Jung, Joo-Hee Park, Ha-Jin Pak, Martha Kim, Roy S. Chuck, Choul Yong Park

**Affiliations:** 1 Department of Ophthalmology, Graduate School of Medicine, Dongguk University, Seoul, South Korea; 2 Department of Ophthalmology, Dongguk University, Ilsan Hospital, 814, Siksadong, Ilsan-dong-gu, Goyang, Kyunggido 410-773 South Korea; 3 Department of Ophthalmology and Visual Sciences, Montefiore Medical Center, Albert Einstein College of Medicine, Bronx, NY USA

**Keywords:** Antibiotics, Translational research, Drug delivery, Eye diseases, Lens diseases

## Abstract

Intraocular antibiotic delivery is an important technique to prevent bacterial infection after ophthalmic surgery, such as cataract surgery. Conventional drug delivery methods, such as antibiotic eye drops, have limitations for intraocular drug delivery due to the intrinsic barrier effect of the cornea. Therefore, frequent instillation of antibiotic eyedrops is necessary to reach a sufficient bactericidal concentration inside the eye. In this study, an intraocular implant, *MXF-HA*, that combines hyaluronic acid (HA) and moxifloxacin (MXF) was developed to increase the efficiency of intraocular drug delivery after surgery. MXF-HA is manufactured as a thin, transparent, yellow-tinted membrane. When inserted into the eye in a dry state, MXF-HA is naturally hydrated and settles in the eye, and the MXF contained therein is delivered by hydrolysis of the polymer over time. It was confirmed through in vivo experiments that MXF delivery was maintained in the anterior chamber of the eye at a concentration sufficient to inhibit *Pseudomonas aeruginosa* and *Staphylococcus aureus* for more than 5 days after implantation. These results suggest that MXF-HA can be utilized as a potential drug delivery method for the prevention and treatment of bacterial infections after ophthalmic surgery.

## Introduction

In recent decades, the rapid growth of the old-age population has become a global health problem. Cataracts is one of the most common health problems in old age. Although there were 10.8 million blind people with cataracts worldwide in 2010, this number will increase to 40 million by 2025, as expected by the World Health Organization^1^. Cataract surgery is the most common procedure performed by ophthalmologists. It is estimated that more than 20 million cataract surgeries are performed worldwide annually^1^. The successful outcome of cataract surgery depends on the surgeon’s skill and postoperative care. It has been believed that ocular hygiene and frequent instillation of antibiotic eyedrops are essential to prevent bacterial ocular infection after surgery, which can lead to blindness. However, recent studies have raised concerns about the effectiveness of topical antibiotics as a preventive method for bacterial endophthalmitis after cataract surgery^2–4^. Instead, an intracameral antibiotic injection at the conclusion of cataract surgery has been proposed as a promising alternative for endophthalmitis prophylaxis^5,6^. Intracameral bolus injection of vancomycin can exceed the minimum inhibitory concentration (MIC) of Gram positive bacteria for up to 26 h^7^. An intracameral injection of moxifloxacin (MXF) exceeded the MIC of *Streptococcus pneumonia, Streptococcus viridans*, fluoroquinolone-susceptible coagulase-negative *Staphylococcus,* and fluoroquinolone-susceptible *Staphylococcus aureus* for 6 h in aqueous humor with a half-life of 2.2 h^8^. 

Fluoroquinolones are the most commonly used antibiotics for the prevention of bacterial endophthalmitis after cataract surgery. Compared with other commercial fluoroquinolones, fourth generation ophthalmic quinolones such as MXF have the broadest spectrum antibiotic activity against most keratitis and endophthalmitis isolates^9^. Improvement in antibiotic potency of the fourth generation quinolones is achieved through its structural modification. Nitrogen at the R8 position improved activity against anaerobes and 2,4-difluorophenyl group at N position improves the overall potency of the drug. These modifications can be seen in the structure of moxifloxacin and travofloxacin^10^. MXF showed better ocular tissue penetration than other fluoroquinolones because it is more water soluble at a physiologic pH of 7.0 and has both hydrophilic and lipophilic properties^9^. In a rabbit model, a single dose topical administration of 0.3% MXF reached a concentration of 1.8 μg/mL in aqueous humor after 30 min^11^. This concentration is 30 times higher than the MIC_90_ of *Staphylococcus epidermidis*.

Hyaluronic acid (HA) is a linear polysaccharide with unique viscoelasticity, biodegradability and biocompatibility^12^. In the field of ophthalmology, HA has long been used as the main substrate of artificial tears, eyedrops, and viscoelastic devices during cataract surgery. The potential of HA as an effective drug carrier for ocular diseases has also been investigated, such as viscous solutions, hydrogels, nanoplatforms, microneedles, and ocular inserts^12–15^.

Mental or physical impairments frequently found in the old-age population may impede the use of eyedrops for post-cataract surgery management. No matter how effective a drug may be, good compliance is a prerequisite for successful topical antibiotic treatment. By inserting a drug delivery system that can continuously secrete antibiotics into the eye at the conclusion of cataract surgery, it is possible to effectively prevent ocular bacterial infections without worrying about compliance with eye drops. To overcome these challenges, we developed an intraocular implant, *MXF-HA*, that can effectively release MXF over a sufficient period by combining HA and MXF. In this study, we analyzed the physical properties and effectiveness of MXF-HA in both in vitro and in vivo settings.

## Results

### Characterization of MXF-HA

#### Water absorption capacity and optical transmittance

**Figure 1:**
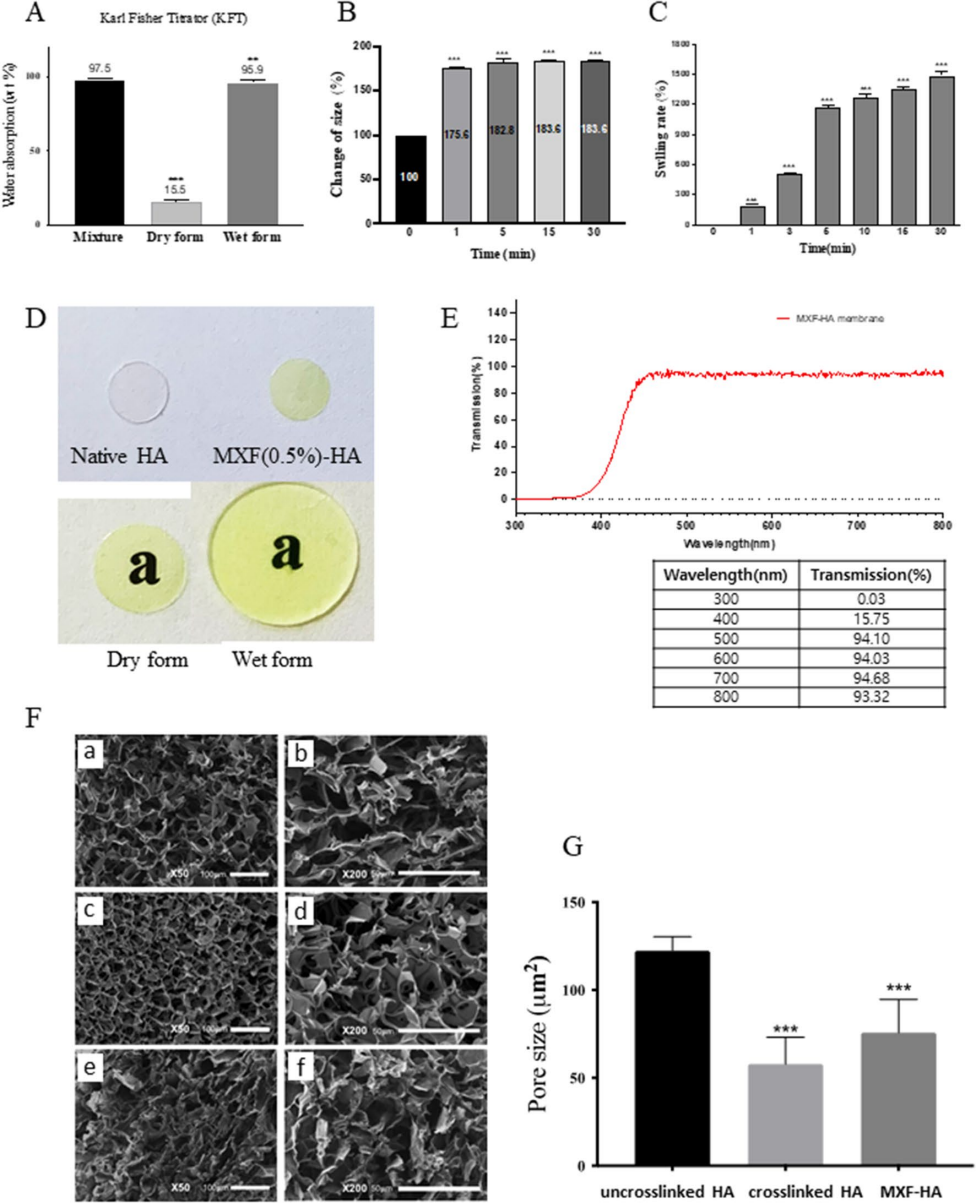
Physical characteristics of the intraocular implant, MXF-HA. (**A**) The water content of the dry form of MXF-HA was measured as 15.5%, while the water content after full hydration was 95.9%. (**B**) The size of MXF-HA increased to about 180% after full hydration was reached (30 min after hydration). (**C**) The weight of MXF-HA increased to about 1500% after full hydration. (**D**,**E**) MXF-HA has excellent transparency with a yellow color and has light transmittance over 93% for light with wavelength 500–800 nm. (**F**,**G**) Examination with a scanning electron microscope revealed a well-organized structure with high porosity and smaller pore size in MXF-HA compared with uncross-linked hyaluronic acid (HA). (a,b) Uncross-linked HA. (c,d) Cross-linked HA. (e,f) MXF-HA. Scale bars indicate 100 μm in (a), (c), and (e) and 50 μm in (b), (d), and (f). **p < 0.01, ***p < 0.001.

MXF-HA was manufactured in a dry form and easily inserted into the anterior chamber of the eye, where it was rapidly hydrated by aqueous humor. The water content of dry-form MXF-HA was 15.5%, but when fully hydrated, the water content reached 95.9% at the equilibrium state in saline (Fig. 1A). The swelling rate of MXF-HA was measured by comparing the size and weight of a 5 mm diameter circle of MXF-HA before and after hydration. Complete hydration took 5 min and the size of MXF-HA increased by 1.8 times compared with the dry state (Fig. 1B). The weight of MXF-HA showed a rapid increase (1200%) up to 5 min after hydration. After that, it gradually increased to about 1500% after 30 min (Fig. 1C). The tint of MXF-HA was light yellow, but it had good enough transparency to clearly read the letters underneath the membrane (Fig. 1D). The optical transmittance of hydrated MXF-HA was measured at different wavelengths (300–800 nm) using a UV–Vis scanning spectrometer (Fig. 1E). The transmittance of MXF-HA was over 93% for light with a wavelength of 500–800 nm, but it fell to about 15% for light with a wavelength under 400 nm.

#### Scanning electron microscope (SEM)

The cross-sectional microscopic morphology of MXF-HA was obtained using a scanning electron microscope (SEM). We analyzed SEM images under the same magnification conditions (× 50, × 200, Secondary Electron Imaging [SEI], and 15 kV) to compare the microstructures of uncross-linked HA, cross-linked HA, and MXF-HA. Cross-linked HA and MXF-HA showed well-organized structures with high porosity and small pore size compared with uncross-linked HA (Fig. 1F,G). However, it is to be noted that the addition of MXF to cross-linked HA to prepare MXF-HA slightly increased the pore size compared with cross-linked HA.

#### Drug-release rate in vitro

**Figure 2:**
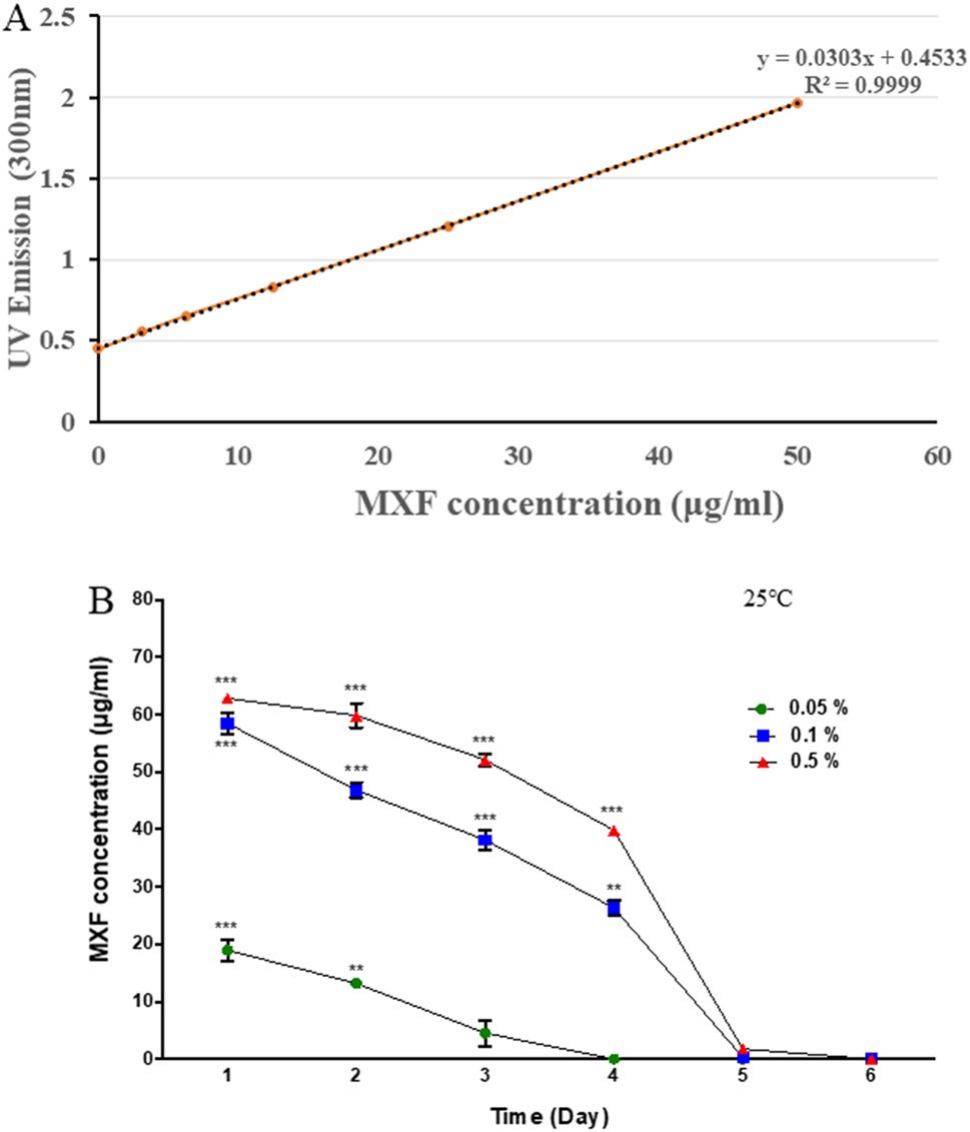
Moxifloxacin (MXF) release from MXF-HA. (**A**) The standard release curves for MXF-HA in saline were obtained by UV–Vis spectrometer at 300 nm. (**B**) The difference in the release pattern according to the concentration of MXF (0.05%, 0.1%, and 0.5%) contained in the preparation of MXF-HA was measured. Significant MXF release was observed by day 4 with 0.1% and 0.5% MXF-HA, whereas measurable MXF release with 0.05% MXF-HA lasted for only 3 days. **p < 0.01, ***p < 0.001.

The calibration curves for MXF-HA in saline are shown in Fig. 2A. The total release of MXF from a 5 mm diameter circle of MXF-HA measured using the standard curve at 300 nm was 254.7 μg (Fig. 2A). The drug loading efficiency of 0.5% MXF-HA was calculated to be 89.4%, since 285 μg of MXF was initially used in its fabrication.

In order to examine whether there is a difference in the release pattern depending on the concentration of MXF contained, MXF-HA was prepared by including three concentrations of MXF (0.05%, 0.1%, and 0.5%) and was tested at 25 °C. The release profiles of MXF-HA showed a similar pattern of initially having a high release rate and later slowing down (Fig. 2B). Significant MXF release was observed by day 4 with 0.1% and 0.5% MXF-HA, whereas measurable MXF release with 0.05% MXF-HA only persisted for 3 days. From these results, it was confirmed once again that it is more effective to use 0.5% MXF for MXF-HA production.

The time required for the complete degradation of MXF-HA was measured to be approximately 48 h in the presence of hyaluronidase and approximately 96 h in the absence of hyaluronidase (Supplementary Fig. 1).

#### Quantification of MXF released from MXF-HA implanted into rabbit eyes

**Figure 3:**
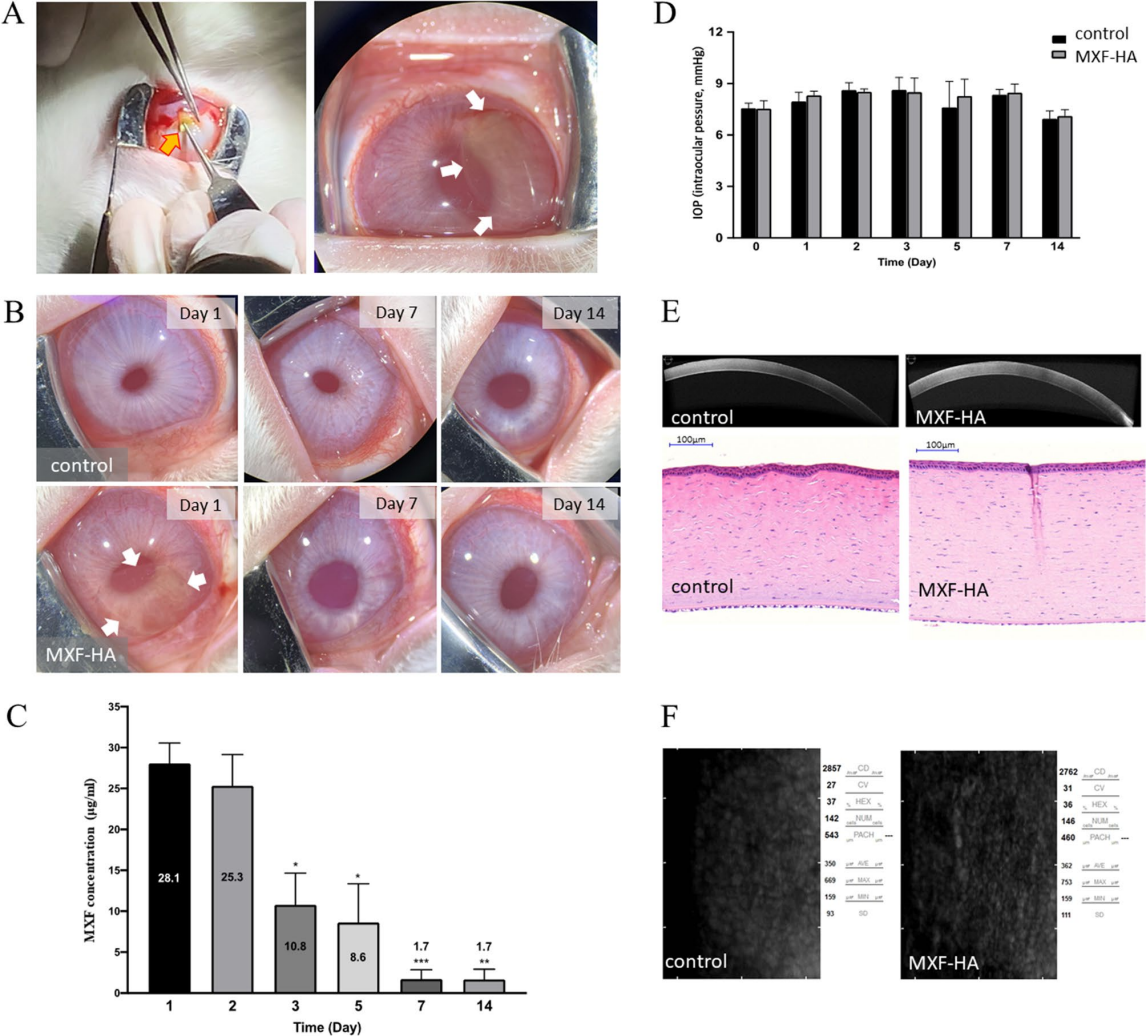
Evaluation of MXF-HA efficacy using a rabbit model. (**A**) MXF-HA is inserted into the anterior chamber through a corneal incision while folded by forceps (yellow arrow). The unfolded circular MXF-HA is visible inside the anterior chamber immediately after implantation (white arrows indicate the margin of MXF-HA). (**B**) The implanted MXF-HA was visible on day 1 after surgery (white arrows). However, no visible MXF-HA structures were detected on days 7 and 14 after the surgery. Control is the contralateral eye without MXF-HA insertion. (**C**) MXF concentration was measured using aqueous humor collection. MXF concentrations in aqueous humor were maintained above 8 μg/mL for 5 days. (**D**) The intraocular pressure of MXF-HA-implanted eyes was not significantly different from that of the contralateral control eyes and was maintained within the normal range. (**E**) Eyeballs were harvested and imaged using optical coherence tomography (OCT). Normal corneal structure and thickness were maintained in both MXF-HA and control eyes. Histology (hematoxylin and eosin [H&E] staining) showed normal cellularity in all corneal layers without signs of inflammation in both MXF-HA and control eyes. (**F**) Specular microscopy revealed normal density and morphology of corneal endothelial cells in both MXF-HA and control eyes. *p < 0.05, **p < 0.01, ***p < 0.001.

MXF-HA is foldable and can be inserted through a small corneal incision. MXF-HA was yellow in color and could be identified in the anterior chamber after implantation. In addition, due to its high transparency, intraocular structures such as the lens or iris could be observed after implantation (Fig. 3A). However, MXF-HA was not visible due to dissolution when observed under a microscope on days 7 and 14 after implantation (Fig. 3B). Nevertheless, the time-dependent MXF release from MXF-HA in the aqueous humor of rabbits is observed. The concentration of MXF in the aqueous humor was maintained above 8 μg/mL for 5 days (Fig. 3C).

There was no significant intraocular pressure (IOP) difference between the control and MXF-HA-implanted eyes (Fig. 3D). Histology and corneal optical coherence tomography (OCT) showed intact corneal structure in MXF-HA-implanted eyes (Fig. 3E). Compared with the control eyes, corneal endothelial cell density and cell morphology, and corneal thickness did not reveal any significant changes in MXF-HA-implanted eyes (Fig. 3F; Table 1).

**Table 1:** Comparison of corneal endothelial cell density and morphology between MXF-HA-implanted and control eyes in rabbits. Data was described as mean ± standard deviation, and *p* values were calculated using the Mann–Whitney test.

	MXF-HA	Control	P-value
Cell density (cells/mm^2^) (range)	2804.5 ± 243.3 (2451–3003)	2801.5 ± 55.8 (2762–2841)	0.533
Hexagonality (%) (range)	42.00 ± 5.03 (37–49)	46.00 ± 14.14 (36–56)	1.000
Corneal thickness (μm) (range)	497.75 ± 30.98 (475–543)	494.00 ± 48.08 (460–528)	0.800

The corneal endothelial safety of MXF-HA was further verified in an in vitro cell viability analysis using immortalized human corneal epithelial cells (Supplementary Fig. 2).

#### Antibiotic effects of extracts from MXF-HA inserted into the whole eye of rats

**Figure 4:**
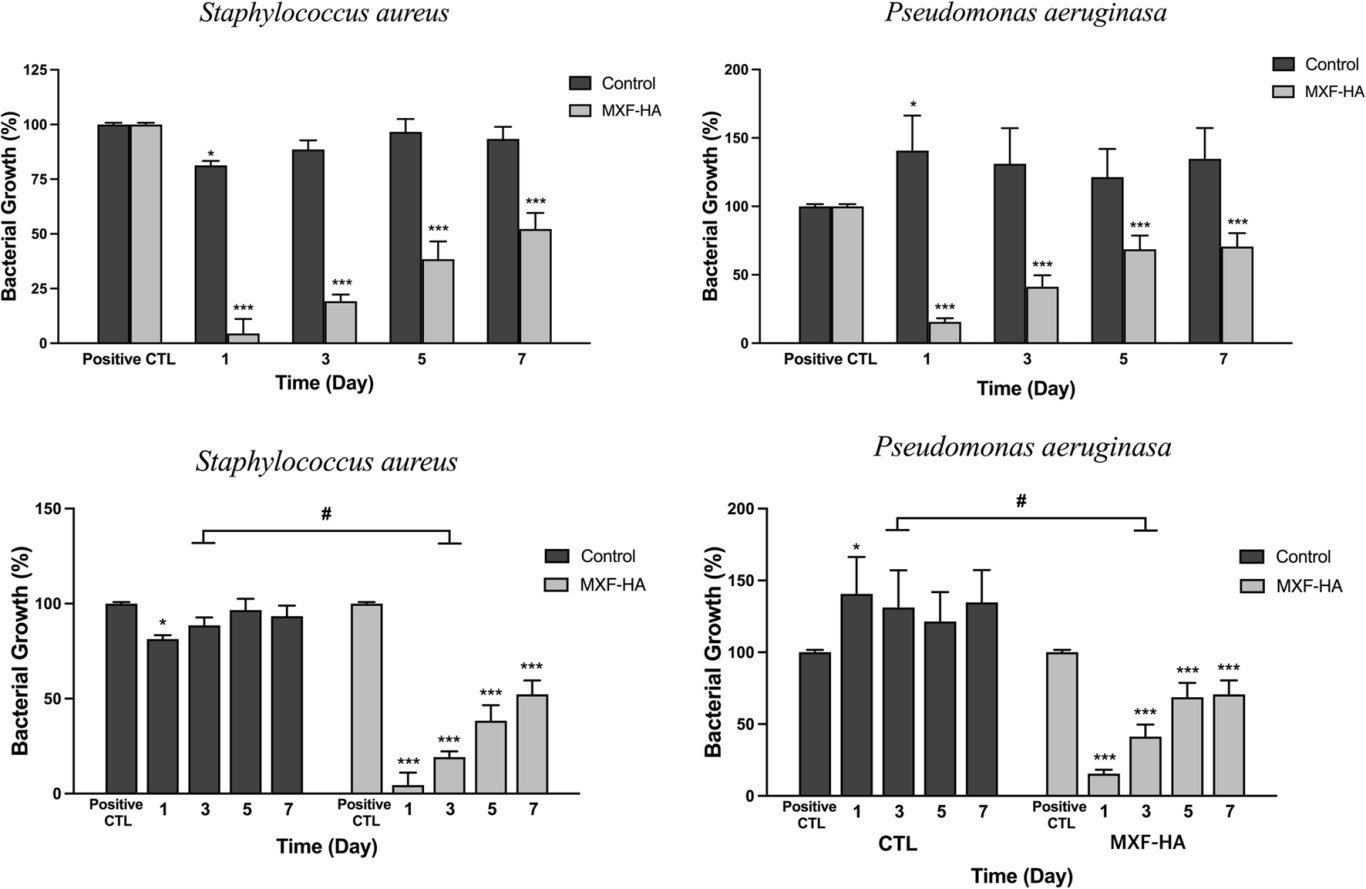
Evaluation of MXF-HA efficacy using a rat model. The extracts from the whole eye of MXF-HA-injected rats effectively inhibited the growth of *Pseudomonas* *aeruginosa* and *Staphylococcus aureus* on the first day of implantation. Statistically significant inhibition of bacterial growth was also observed in experiments with rat whole eye extracts collected 7 days after MXF-HA implantation. The positive control (positive CTL) represent the left eyes and right eyes of non-treated rats at day 0. *p < 0.05, ***p < 0.001.

MXF-HA-injected rat eye extract effectively inhibited the growth of *Pseudomonas aeruginosa and S. aureus* on the first day of implantation. However, this effect gradually weakened thereafter (Fig. 4 and Supplementary Fig. 3). This may have been due to technical difficulties, so the antibacterial activity was evaluated based on the MXF concentration in the entire eye extract, not the MXF concentration in the anterior chamber. In rats, the volume ratio of the anterior chamber to the entire eye in rats is less than 10%, so it is expected to be more effective than the antibacterial effect indirectly demonstrated by our experiment. In addition, since the bacterial inhibition experiment was conducted by adding the eye extract (10 μL) to the bacterial culture medium (3 mL), it cannot be overlooked that there is a 300-fold dilution effect in the experimental technique.

## Discussion

In this study, we developed a sustained MXF-releasing HA membrane by cross-linking with 1,4-butanediol diglycidyl ether (BDDE). The optical transparency of MXF-HA was excellent, with good penetration of the visible range of light. MXF-HA was manufactured as a thin membrane and round punched (diameter 5 mm) in this study to deliver high enough concentrations of MXF for common pathogen inhibition. The MXF was released for up to 5 days after intraocular implantation above the MIC_90_ of *S. aureus*. Intraocular safety for corneal endothelial cells was verified, and the IOP was maintained in the normal range until the dissolution of MXF-HA.

Previously we developed a BDDE cross-linked HA membrane for ocular surface treatment^16^. In our previous report, the residual BDDE in the HA membrane was quantified as being below the Food and Drug Administration safety limit of 2 parts per million (ppm) for safe human application^16^. We verified the safety and efficacy of cross-linked HA membranes for corneal epithelial cell proliferation, corneal wound healing after mechanical or chemical injury and conjunctival wound healing^16^. In order to further expand the use of HA membranes, the drug loading potential was evaluated and some positive results were observed through this study.

The reason that we selected MXF and HA as the main materials is that these two materials are already widely used in the ophthalmology field and safety has already been supported by decades of clinical experience. HA is a well investigated polymer in the ophthalmic field. HA is the highest-selling artificial tear ingredient^17^. The epithelial healing and anti-inflammatory effect of HA has been repeatedly reported^18,19^. HA is an essential component of ophthalmic viscoelastic devices, which maintain the anterior chamber during cataract surgery^20^. The unique physical characteristics of HA, such as viscoelastic and mucoadhesive capacities, facilitated the research to develop HA as an effective vehicle for topical ophthalmic drugs. The extension of precorneal residence time by HA can increase the bioavailability of the conjugated drug component^21,22^. Recently, research to develop HA as an intravitreal drug delivery material for the treatment of age-related macular degeneration is being actively conducted^12^. However, studies using HA in the form of an intraocular drug implant after cataract surgery are limited^23^.

There is a risk that elevated IOP can be caused by the retained HA in routine cataract surgery. It is practically impossible to completely remove all the intraocularly injected HA during cataract surgery due to the intrinsic nature of this material. However, if it remains above a threshold level, HA can block the trabecular meshwork pathway for aqueous drainage, which can result in an acute rise in IOP. This is why we monitored IOP in rabbit eyes with MXF-HA implantation. We confirmed normal maintenance of IOP in MXF-HA-implanted rabbit eyes at least until day 14.

The sustained release of MXF from MXF-HA is one of the most interesting findings of this study. As SEM images revealed, MXF-HA has a homogenous and rigid structure with a high density of small pores. The morphological structure and interconnectivity were known to depend on the strength of the cross-linking^24^. Our hypothesis is that MXF is captured in these pores during the manufacturing process, and the slow destruction of the honeycomb structure of MXF-HA can release the MXF sequestered inside the pores. It was reported that the cross-linking process can increase the molecular chains of HA and weaken water solubility; thus, mechanical strength is increased with a prolonged degradation time^12^. The duration of drug release from MXF-HA is proportional to degradation rate of the polymer. In a preliminary study, we found that the higher the concentration of MXF mixture, the faster the rate of MXF-HA degradation. For the clinical applicability of MXF-HA, 0.5% MXF was selected as the best choice for its antibacterial activity and duration of drug release.

The finding of at least 5 days of maintenance of the MIC_90_ of *S. aureus* after MXF-HA implantation is promising. It is known that intracameral antibiotic bolus injections can last for several hours up to 26 h^7^. Recently, most phacoemulsification cataract surgery is performed through a small clear corneal incision (sized 2.2–3.5 mm) without any suture^25,26^. Intentional hydration techniques (hydro-sealing techniques) of the main wound at the conclusion of the surgery can maintain the anterior chamber without any suture. However, this clear corneal incision can leak immediately after the cataract surgery, and a gentle focal pressure can reopen clear corneal incisions even several days after the surgery^27^. Therefore, it is necessary to use prophylactic antibiotics to prevent bacterial infection through the opened corneal incision for at least 5 days after cataract surgery. In this respect, our finding of sustained antibiotic release from MXF-HA has potential clinical significance.

MXF is a broad-spectrum antibiotic effective against Gram-positive and Gram-negative bacteria by inhibiting bacterial DNA gyrase and topoisomerase IV which are necessary for bacterial replication^28^. The MIC_90_ of MXF is less than 0.25 mg/L for commonly isolated community acquired respiratory tract pathogens^28^. However, in ophthalmic infections, therapeutic concentrations of MXF are required to be relatively high. It was reported that the MIC_50_ and MIC_90_ of MXF for ciprofloxacin-resistant *S. aureus* were 1 μg/mL and 8 μg/mL, respectively, and the MIC_50_ and MIC_90_ of MXF for *P. aeruginosa* were reported as 1 μg/mL and 128 μg/mL, respectively^29,30^. As evidenced by our in vivo data, the measured MXF concentration at day 5 after MXF-HA implantation was 8.6 μg/mL. This concentration inhibited the growth of *S. aureus* by about 90% for 5 days and *P. aeruginosa* by about 80% for 3 days after the surgery. It is noted that MXF-HA has higher early drug release observed on days 1 and 2 compared with later. We believe that early bolus release may have some advantage in controlling intraoperative bacterial contamination.

The membranous form of MXF-HA has some advantages. Before hydration, the physical properties of MXF-HA mean it is hard enough to be easily inserted into a small corneal incision. However, after hydration, the volume increases and MXF-HA does not escape through the small corneal incision. Therefore, the whole implantation procedure of MXF-HA generally takes less than 1 min and does not prolong the surgical time significantly.

There have been many efforts to develop sustained intraocular drug delivery platforms. In particular, since anti-vascular endothelial growth factor (VEGF) treatment for age-related macular degeneration requires repeated intraocular injections, there are many active investigations to develop a sustained anti-VEGF drug delivery platform with a single injection in order to minimize the need for repeated intraocular injections^31^. There are also studies on drug delivery systems that continuously deliver drugs in the anterior chamber after cataract surgery. In particular, drug loading on the intraocular lens has been actively investigated^32,33^. One study reported an effort to load both antibiotic and anti-inflammatory drugs on an intraocular lens material and had a promising result, with more than 15 days of release of MXF and ketorolac being observed^32^. More than 4 weeks of antibiotic release from a poly(2-hydroxyethyl-methacrylate) (pHEMA) hydrogel intraocular lens combined with norfloxacin during its manufacture was reported^34^. However, there is an important fact that cannot be overlooked. The choice of intraocular lens is a very important decision of the surgeon for better optical results after cataract surgery. If the drug delivery system is incorporated with an intraocular lens, there is a disadvantage in that a specific intraocular lens should inevitably be used for the drug delivery regardless of the patient’s need and surgeon’s preference. In this respect, MXF-HA has a potential advantage since it can be inserted at the conclusion of the surgery regardless of what type of intraocular lens is used. MXF-HA also has advantages in terms of manufacturing cost and ease of manufacture. Apart from antibiotics, there have also been studies to deliver anti-inflammatory drugs using a drug delivery device after cataract surgery. Dexamethasone delivery using PLGA (poly(lactic-glycolic)-acid) polymer (Surodex, Oculex Pharmaceuticals, Inc.) was developed, but it was not successful in the market^35^.

The poor compliance often observed in old-age patients with mental or physical disabilities can increase the risk of bacterial infection after cataract surgery^36–38^. These patients are often unable to control the number of drops for each topical medication, instill an incorrect number of drops, or accidentally instill the wrong eye drops. The contamination of the tip of the eyedrop bottle can also lead to an increased risk of bacterial infection during the vulnerable postoperative period. The potential advantage of MXF-HA is that it can replace the frequent eyedrop instillation required after cataract surgery. The finding of sustained delivery of MXF with a one-time MXF-HA implantation is promising in that it can be useful in the setting of cataract surgery camps in vulnerable communities where intense postoperative medical care is impossible.

Our study has several limitations. The efficacy of MXF-HA was evaluated only for *S. aureus* and *P. aeruginosa*. These two bacteria were selected as common pathogens of many ocular infections, such as keratitis, conjunctivitis, and endophthalmitis^39^. However, the most common pathogen of post-cataract surgery bacterial endophthalmitis is known to be coagulase-negative *Staphylococcus*, such as *S. epidermidis*^3^. Further investigation using various ocular pathogens, including coagulase-negative *Staphylococcus*, *Streptococcus*, and other Gram-negative bacilli, could have enhanced the confidence in the efficacy of MXF-HA. Due to the small volume of rat aqueous humor, MXF release from MXF-HA implanted into rat eyes was indirectly measured using whole eye extract. Therefore, the result can be the antibiotic effect of the entire eyeball rather than that in the anterior chamber. This has a limitation because it is not a direct representation of the antibiotic effect in the anterior chamber, which is an important target in cataract surgery. Limiting the study to MXF among other fluoroquinolone antibiotics is another weakness of this study. It would be more valuable if various other antibiotics were investigated as the combination with HA. Ultimately, since the combined administration of antibiotics and anti-inflammatory drugs is a routine requirement after cataract surgery, a future study should focus on the development of a complex that can deliver both antibiotics and anti-inflammatory drugs at the same time after a single implantation.

In conclusion, as the aging population grows, compliance issues with frequent eye drops after cataract surgery will continue. In this study, MXF-HA, which can be implanted into the eye, was developed to effectively prevent post-operative bacterial infection. MXF-HA is transparent and easy to implant at the conclusion of cataract surgery. Its safety and effectiveness were verified using animal models. Our results show that MXF-HA has the potential to replace conventional postoperative antibiotic eye drop treatment.

## Materials and methods

### Preparation of MXF-HA

**Figure 5:**
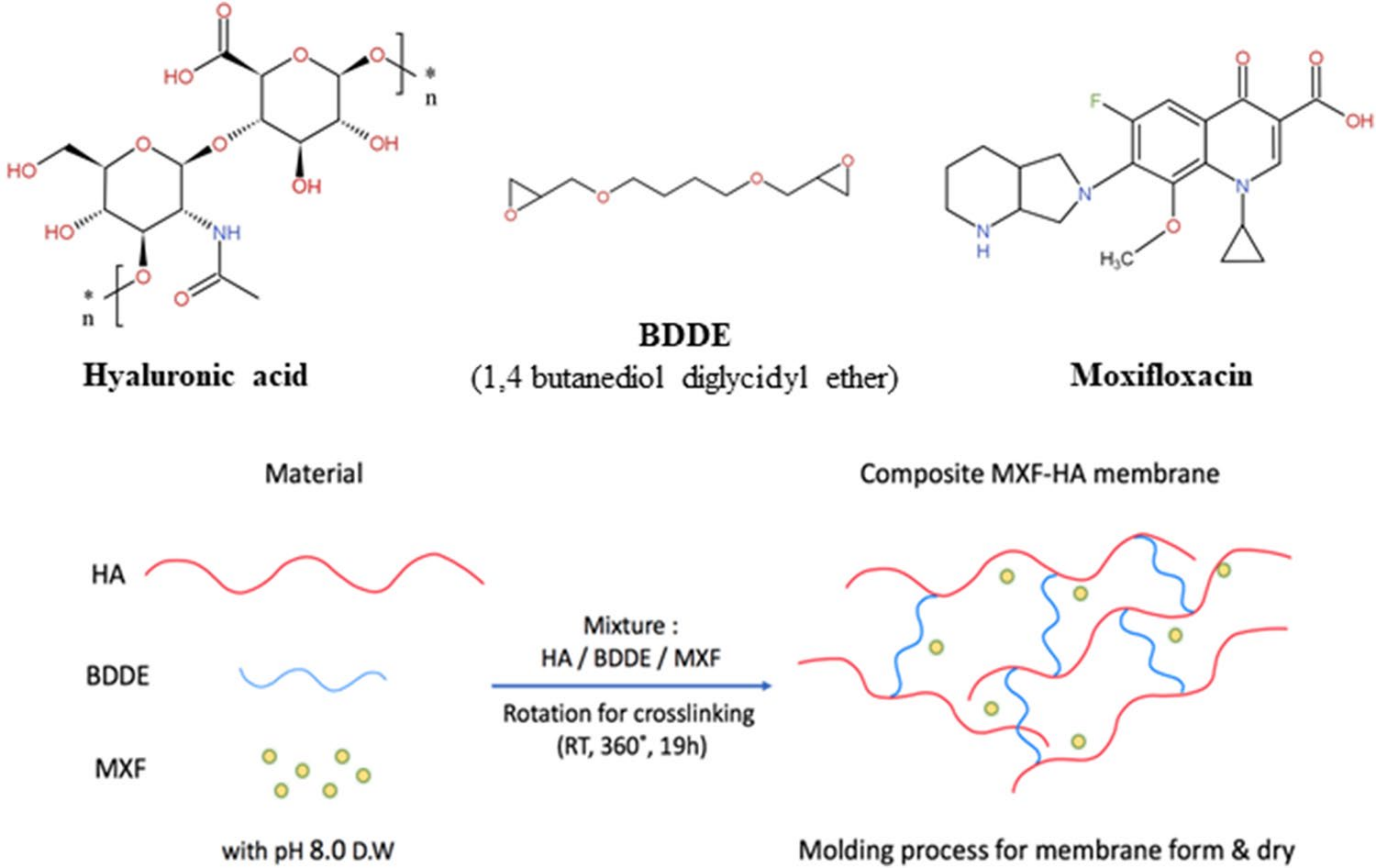
Schematic illustration showing the manufacture of MXF-HA intraocular implant.

MXF-HA was fabricated based on previously established methods^16^. In brief, a hydrogel was manufactured by mixing 3% sodium HA solution (average molecular weight = 700,000 Da, SK Bioland, Cheonan, South Korea) with 1,4-butanediol diglycidyl ether (BDDE, 0.01%, Sigma Aldrich, St. Louis, Missouri, USA) and 0.5% MXF (Sigma Aldrich). BDDE plays the role of the cross-linker for HA. For the cross-linking reaction, the mixture was incubated for 19 h by rotating it 360° at room temperature. Then the mixture was spread onto a plate and dried (24 h of flat stirring and 72 h of drying in the clean bench at room temperature) to form the MXF-HA membrane (Fig. 5). The whole procedure was carried out under aseptic conditions. The corneal endothelial safety of MXF-HA was analyzed using human corneal endothelial cell line (B4G12, Creative Bioarray, Shirley, NY, USA). The cell viability was measured by CCK-8 analysis after culturing cells in MXF-HA containing culture medium (5 mm diameter MXF-HA/1 mL, concentration of MXF 261 μg/1 mL).

### Physical characterization of MXF-HA

#### Swelling ratio and water absorption capacity

Hydrogel absorbs water and maintain its shape in its hydrated state. Round cuts (diameter of 5 mm) of dry MXF-HA were hydrated in normal saline. The diameter and weight of hydrated MXF-HA were measured sequentially at 1, 5, 15 and 30 min. When reaching the equilibrium state, the diameter and weight of MXF-HA no longer changed and the time reaching this state was recorded.

The water content of MXF-HA was measured by Karl-Fischer titration^40^. The coulometric method (Mettler Toledo, Metrohm 852 Titrando Karl Fisher Titrator with Metrohm 874 Oven Sample Processor, Germany) was used for the water content measurement of dry MXF-HA and the volumetric method (Mettler Toledo, Metrohm 852 Titrando Karl Fisher Titrator Metrohm 860 KF Thermoprep, Germany) was used to determine the water content of fully hydrated MXF-HA.

#### Scanning electron microscope (SEM)

The morphology of native HA, cross-linked HA and MXF-HA was visualized by SEM using an IT-500HR instrument (JEOL, Tokyo, Japan) as previously described^16^. All samples were lyophilized prior to processing. Images were taken in a Secondary Electron Imaging (SEI) mode with a voltage of 15 kV. Each sample solution was frozen overnight, then immersed in liquid nitrogen for 1 h, and the samples were fixed to aluminum plates with carbon tape and then coated by platinum sputtering. The pore size of the honeycomb structure of MXF-HA was measured using image analysis software (ImageJ, Scion Corp., Frederick, Maryland, USA).

#### Measurement of optical transmittance

The optical transmittance of hydrated MXF-HA was measured using a Scinco UV–Vis scanning spectrometer (Model S-4100, Scinco, Daejeon, South Korea) as previously described^16^. A round cut of MXF-HA with a diameter of 5 mm was hydrated in normal saline for 5 min. The optical transmittance of MXF-HA was measured in the visible light range. The transparency of MXF-HA was determined by UV–Vis analysis.

#### Enzymatic and hydrolytic degradation rate of MXF-HA

Dry MXF-HA was cut to contain 100 mg of weight and was placed in a cell strainer (Catalog No. SPL93040, 40 μm pore size, SPL life science, Pocheon, South Korea). The cell strainer containing MXF-HA was placed in each well of a 6-well plate containing normal saline with or without hyaluronidase (100 U/mL, Catalog No. 37326-33-3, type I-S hyaluronidase from bovine testes, Sigma Aldrich) at 37 °C. After reaching full hydration after 30 min in solution, the cell strainer-MXF-HA complex was harvested, dried and weighed. The weight of MXF-HA was calculated by subtracting the strainer weight from the total weight. The weight measured at 30 min was used as the control. The cell strainer-MXF-HA complex was then placed back into each well of a 6-well plate containing normal saline with or without hyaluronidase. The weight of partially degraded MXF-HA was measured and calculated at 1, 3, 6, 12, 24, 48, 72, and 96 h.

### Evaluation of drug loading and release

The spectrophotometric method was used to measure the total amount of MXF contained in a 5 mm diameter round cut of MXF-HA^41–43^. MXF is a slightly yellowish crystalline substance that retains its yellow color even in MXF solution. In brief, various concentrations (0–0.5%) of MXF solution were prepared and scanned at various UV wavelengths (300 nm) to determine a standard curve between MXF concentration and UV absorbance. Across the whole range of measured UV wavelengths, the absorbance and MXF concentration showed a linear relationship. The wavelength band of 300 nm was selected as the standard for this study because this was where the highest correlation coefficient was found (Spearman correlation, *R*^2^ = 0.9999, *p* < 0.001). MXF released from MXF-HA in vitro was quantified from day 1 to day 6. A round cut of MXF-HA with a diameter of 5 mm was hydrated in 2 mL of saline. The drug released in saline was sampled daily and evaluated by a UV–Vis spectrophotometer (Spark Multimode Microplate Reader, Tecan Trading AG, Switzerland) at 300 nm, and the MXF concentration was calculated using the standard curve. After each measurement, the old solution was gently discarded and the fresh saline was filled into the chamber with MXF-HA to minimize the effect of previously released MXF. The analysis was repeated three times, and the mean value with standard deviation was reported.

The evaluation of drug loading efficiency was measured using a UV–Vis spectrometer at 300 nm, and the total amount of MXF released was compared with the total amount of MXF used to manufacture MXF-HA. This value was calculated using following equation.

$$ Drug\; loading \;efficiency\; (\% ) = 100 - \frac{Total \;amount \;of \;MXF - Total\; amount \;of\; released \;MXF}{{Total\; amount\; of \;MXF}} \times 100. $$

### Bacterial culture and antimicrobial activity tests

**Figure 6:**
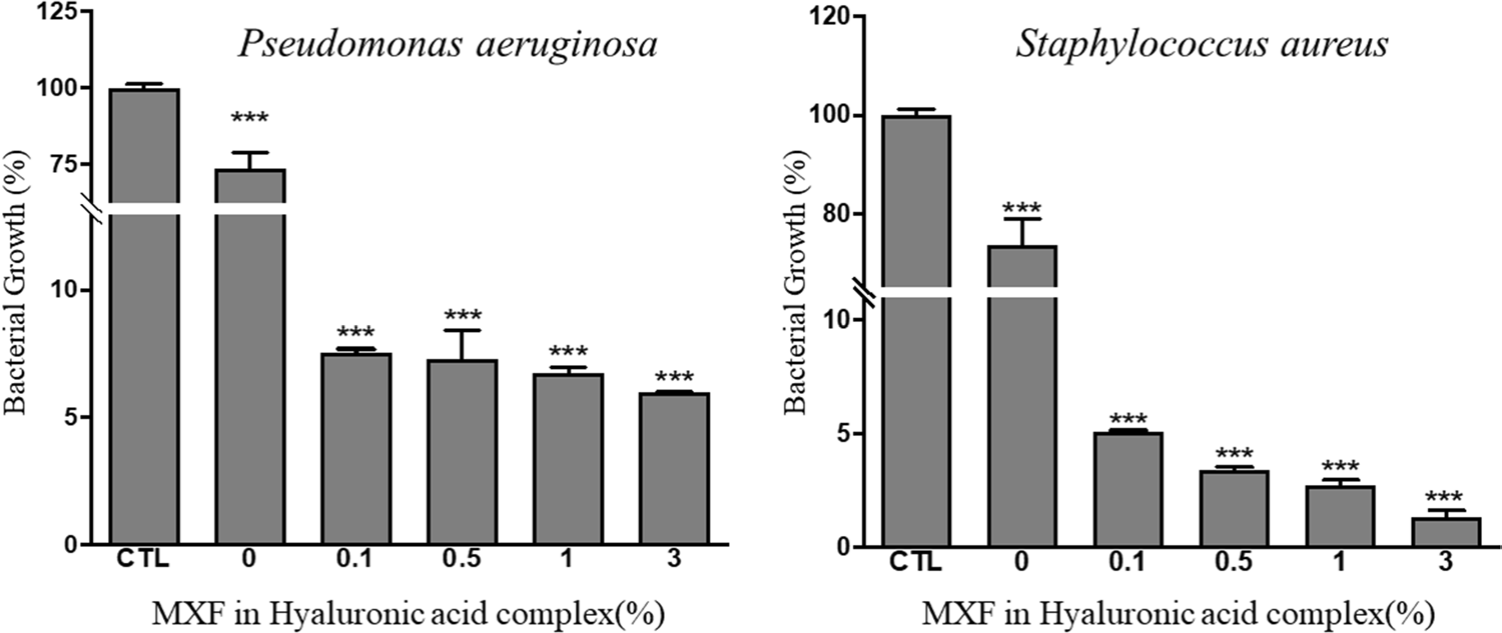
Determination of MXF concentration for MXF-HA manufacture. The growth inhibition of two common ocular pathogens (*P. aeruginosa* and *S. aureus*) was evaluated using various concentrations of MXF. MXF-HA with an MXF concentration of 0.1% or higher inhibited the growth of *P*. *aeruginosa* to less than 10% and the growth of *S*. *aureus* to less than 5% compared with the control. ***p < 0.001.

Two common pathogens of infectious ophthalmic disease, *P. aeruginosa* (ATCC 25923) and *S. aureus* (ATCC 10145), were purchased from the American Type Culture Collection (ATCC; Manassas, Virginia, USA). Both bacteria were grown in a tryptic soy liquid broth (BD Bacto, catalog number 211825). The culture for *P. aeruginosa* and *S. aureus* was incubated in a shaker incubator at approximately 150 rpm at 37 °C. To evaluate the optimal MXF concentration for use in MXF-HA manufacture, MXF-HA was prepared using various concentrations (0–3%) of MXF. MXF-HA (5 mm diameter round cut) and were co-incubated with 3 mL of bacterial solution *(S. aureus* or *P. aeruginosa*) at 37 °C for 24 h. Then, the optical density of the bacterial solution was measured at a wavelength of 600 nm using a spectrophotometer (Spark Multimode Microplate Reader, Tecan Trading AG, Switzerland). It was found that MXF-HA with an MXF concentration of 0.1% or higher could inhibit the growth of *P. aeruginosa* to less than 10% and the growth of *S. aureus* to less than 5% compared with the control (0% MXF) (Fig. 6) Thus, 0.5% of MXF was selected for the final MXF-HA product because this concentration is the maximum that can be dissolved without adding DMSO.

### In vivo study

Male, specific pathogen-free (SPF), New Zealand White rabbits (n = 4, 2.0–2.5 kg) were purchased from Samtako Bio Korea (Osan, South Korea), and male Sprague–Dawley rats (n = 20, 12 weeks old) were purchased from Koatech (Gyeonggi-do, South Korea).

Animals were treated in compliance with the Association for Research in Vision and Ophthalmology (ARVO) Statement for the Use of Animals in Ophthalmic and Vision Research and the Animal Research Reporting of In Vivo Experiments (ARRIVE) guidelines. The experimental protocol was approved by the Institutional Animal Care and Use Committee (IACUC) of Dongguk University, Ilsan Hospital, South Korea (reference number 2021-04215).

#### Quantification of MXF released from MXF-HA implanted into rabbit eyes

After systemic anesthesia using Zoletil (Virbac Corporation) and topical anesthesia (Alcaine, Alcon Laboratory, Fort Worth, Texas, USA), the IOP of the rabbits was measured using a rebound tonometer (iCare, Vantaa, Finland). Then, a scleral tunnel incision with a size of 3.0 mm × 3.0 mm was made using a disposable keratome in their right eyes under a surgical microscope. MXF-HA (5 mm diameter round cut) was folded with McPherson forceps and inserted into the anterior chamber of the right eyes. After confirming the natural spread of MXF-HA in the anterior chamber, leakage from the incision site was examined. The left eyes were used as controls. It was confirmed that there was no leakage from the wound without sutures. No antibiotics were prescribed after the surgery. The eyes of the rabbits were examined on postoperative days 0, 1, 7, and 14. Anterior segment photography and anterior chamber paracentesis for aqueous humor collection were performed on each examination day. IOP measurements were performed on days 0, 1, 2, 3, 5, 7, and 14 in both MXF-HA-implanted eyes and control eyes. The collected aqueous humor was diluted, and quantification of MXF was performed using a UV–Vis spectrophotometer (Spark Multimode Microplate Reader, Tecan Trading AG, Switzerland) at 300 nm based on the previously established standard curve.

On the last day (day 14), the rabbits were euthanized after the examination. Using freshly harvested eyeballs (within 1 h after death), corneal thickness and endothelial cell density were measured by specular microscopy (Konan Medical Inc., Japan). Anterior segment optical coherence tomography (OCT) (PLEX Elite 9000, Zeiss, Germany) images were also taken. Finally, the tissues were fixed with 10% formalin, embedded in paraffin, and sectioned (4 μm). Histologic evaluation was performed with hematoxylin and eosin (H&E) staining.

#### Antibiotic effects of extracts from MXF-HA inserted rats’ eyes

After systemic anesthesia using Zoletil (Virbac Corporation) and topical anesthesia (Alcaine, Alcon Laboratory, Fort Worth, Texas, USA), a 1.0 mm-sized corneal incision was made in the right eyes of the rats using a disposable corneal blade. MXF-HA (0.5%, 3.0 mm × 0.5 mm sized, rectangular shape) was inserted into the anterior chamber. This size of MXF-HA contains about 20 μg of MXF. Assuming that the intraocular volume of a 12-week-old rat is 50 μL, the total MXF concentration injected into rat eyes was 400 μg/mL^44^.

The rats were randomly divided into four groups (n = 5 in each group). Each group was sacrificed on days 1, 3, 5, and 7 respectively after MXF-HA implantation. The amount of aqueous humor in rats is so small that it is technically difficult to collect. Therefore, the concentration of MXF in aqueous humor could not be measured directly, but instead, the presence of MXF was indirectly confirmed by proving the antibacterial effect of the extraction solution by grinding the entire rat eye. After the sacrifice, eyeballs were harvested, ground and centrifuged at 14,500 rpm for 15 min at 4 °C, and then the supernatant was obtained. *P. aeruginosa* and *S. aureus* were cultured for 24 h in tryptic soy liquid broth (BD 211825) in advance. The supernatant (10 μL) was added to the culture media (3 mL). After 24 h, the optical density of the bacterial solution was measured at a wavelength of 600 nm using a spectrophotometer (Spark Multimode Microplate Reader, Tecan Trading AG, Switzerland) and the colony forming assay was performed. For colony forming assay, microbial suspension were applied to Petrifilm™ Aerobic Count (AC) plates (3 M™, Saint Paul, MN, USA) at 37 °C for 48 h and CFU count was performed using MediXgraph CFU scope v1.4 software.

### Statistics

Data are presented as mean ± standard error, and statistical significance was determined using a one-way analysis of variance (ANOVA), followed by Dunnett’s multiple comparison test. In this study, statistical significance was expressed as asterisks using New England Journal of Medicine formatting for *p* values, and calculations were completed with GraphPad Prism Ver. 7.01 (GraphPad Software, Inc., La Jolla, California, USA).

## Supplementary Information

Supplementary Figures.

## Data Availability

The datasets generated during and/or analysed during the current study are available from the corresponding author on reasonable request.
