# Supplementary material for: Moxifloxacin releasing intraocular implant based on a cross-linked hyaluronic acid membrane
Source: Sci Rep. 2021 Dec 16;11:24115. doi: 10.1038/s41598-021-03605-0 (PMC8677739; doi:10.1038/s41598-021-03605-0)

Moxifloxacin releasing intraocular implant based on a cross-linked hyaluronic acid membrane

Dong Ju Kim, MD^1^, Mi-Young Jung^2^, Joo-Hee Park, PhD^2^, Ha-Jin Pak MS^2^, Martha Kim MD, PhD^2^, Roy S. Chuck MD, PhD^3^, Choul Yong Park MD, PhD^2^

1. Department of Ophthalmology, Graduate School of Medicine, Dongguk University, Seoul, South Korea
2. Department of Ophthalmology, Dongguk University, Ilsan Hospital, Goyang, South Korea
3. Department of Ophthalmology and Visual Sciences, Montefiore Medical Center, Albert Einstein College of Medicine, Bronx, NY USA

Dong Ju Kim (pursuit4@naver.com), Mi-Young Jung (myjung202@gmail.com), Joo-Hee Park ([asalella00@gmail.com](mailto:asalella00@gmail.com)), Ha-Jin Pak ([sir_campbell@naver.com](mailto:sir_campbell@naver.com)), Martha Kim (marthakim22@gmail.com), Roy S. Chuck(rchuck@montefiore.org), Choul Yong Park (oph0112@gmail.com)

The authors have no proprietary interest in the materials presented herein.

*Correspondence to:

Choul Yong Park MD, PhD

Department of Ophthalmology

Dongguk University, Ilsan Hospital

814, Siksadong, Ilsan-dong-gu, Goyang

Kyunggido, South Korea, 410-773

Tel: 82-31-961-7395

Fax: 82-31-961-7977

Email: [oph0112@gmail.com](mailto:oph0112@gmail.com)

**Supporting information**

**Supplementary Figure 1.**

The degradation rate of MXF-HA was measured at 37 ºC. Hyaluronidase (100U/ml) was used for the enzymatic degradation and normal saline was used for the hydrolytic degradation. The dry weight of MXF-HA remaining after degradation was calculated as a percentage compared to the dry weight of MXF-HA measured at 0.5h. Enzymatic degradation is faster than hydrolytic degradation. **p < 0.01, ***p < 0.001, ^#^p<0.05, ^##^p<0.01, ^###^p<0.001

**Supplementary Figure 2.**

The viability of human corneal endothelial cell (immortalized human corneal endothelial cell, B4G12) in cultures mixed with MXF-HA was analyzed. Round cut MXF-HA (diameter 5mm) was added to 1ml of culture medium in each well of a 24-well plate. We found a subtle decrease in cell viability at 48 and 72 h, but overall viability was maintained over 90% compared to the control (medium without MXF-HA). ***p < 0.001

**Supplementary Figure 3.**

The antibacterial effect of MXF-HA implantation in rat eyes was evaluated using colony counting assay. We confirmed the effect of MXF-HA up to 7 days. A: Representative pictures of colony growth in the plates. B: Colony counting using ImageJ software (version 2.0) demonstrated the significant antibacterial effect against P. aeruginosa and S. aureus until day 7 after MXF-HA implantation in rat eyes. CTL: control with no addition of rat eye extract. ***p < 0.001


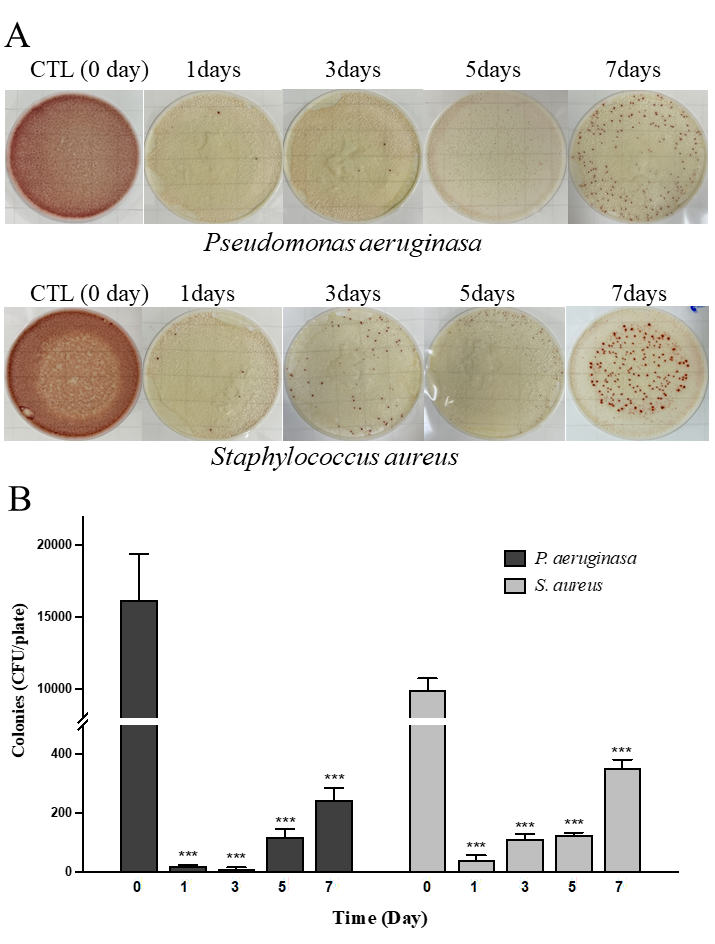

Supplement: Supplementary file 1 — Supplementary Figures. [file 41598_2021_3605_MOESM1_ESM.docx]
